# Human closed and open apex premolar teeth express different toll‐like receptor

**DOI:** 10.1002/mgg3.1268

**Published:** 2020-05-13

**Authors:** Reza Jafari, Razieh Karamzadeh, Faezeh Pesaran Hajabbas, Fereshteh Sayyadizadeh, Zahra Chekini, Samaneh Aghajanpour, Leila Shakeri, Kiumars Nazarimoghaddam, Reza Aflatoonian

**Affiliations:** ^1^ School of Medicine Shahroud University of Medical Sciences Shahroud Iran; ^2^ Department of Endocrinology and Female Infertility Reproductive Biomedicine Research Center Royan Institute for Reproductive Biomedicine ACECR Tehran Iran; ^3^ Department of Stem Cell and Development Biology at Cell Science Research Center Royan Institute for Stem Cell Biology and Technology ACECR Tehran Iran; ^4^ Faculty of Dentistry Shahed University Tehran Iran; ^5^ Faculties of Dentistry Tehran Islamic Azad University Tehran Iran; ^6^ Anatomy Department School of Medicine Iran University of Medical Science Tehran Iran

**Keywords:** dental pulp, inflammation, innate immunity, TLR, tooth apex

## Abstract

**Background:**

The innate immune activation which promotes inflammation responses in the dental pulp tissue leads to the progression of dentin caries. Accordingly, toll‐like receptors (TLRs) are key molecules of the innate immune system that identify pathogen‐associated molecular patterns (PAMPs) on microorganisms and may have a critical role in a dental injury. Therefore, this study aimed to investigate the expression of *TLR2*, *TLR3*, and *TLR4* in the human dental pulp of opened and closed apex teeth.

**Methods:**

Human dental pulps were derived from the healthy opened and closed apex premolar, in which extraction was indicated for orthodontic reasons. The extraction of RNA was performed and the gene expression determined by real‐time polymerase chain reaction (RT‐PCR). The result from real‐time PCR was confirmed using western blot analysis.

**Results:**

Real‐time PCR data analysis showed that the expression *TLR2* and *TLR4* were significantly increased in closed apex premolar teeth compared to open apex teeth, whereas *TLR3* expression was not significantly different in these two groups *(p* < .05).

**Conclusion:**

The results of the present study suggested increased expression of *TLR2* and *TLR4* by the maturation of the apex, which may be due to the presence of microorganisms in the normal or destructed dental pulp tissue. Thus, identifying the expression of TLRs molecules in dental pulp tissue helps to develop a deeper knowledge of the immune responses in the oral cavity.

## Graphical abstract

This study aimed to investigate the expression of TLR2, TLR3, and TLR4 in the human dental pulp of opened and closed apex teeth. The results of the present study suggested increased expression of TLR2 and TLR4 by the maturation of the apex, which may be due to the presence of microorganisms in the normal or destructed dental pulp tissue. Thus, identifying the expression of TLRs molecules in dental pulp tissue helps to develop a deeper knowledge of the immune responses in the oral cavity. Mean ± SEM of normalized gene expression values for TLR2‐4 (above) and western blotting analysis for detection of TLR2‐4 (below).

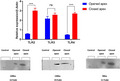

## 1 INTRODUCTION

The dentin is the main component of the tooth organ that is supported by a complex tissue secreting called dental pulp (Goldberg, Kulkarni, Young, & Boskey, 2011). The pulp‐dentin complex consists of many cells such as immune cells, fibroblasts, vascular cells, mesenchymal progenitor cells, and nerve cells (Friedlander, Cullinan, & Love, 2009). Dental pulp cells identify pathogens and activate the innate and/or adaptive immunity to combat pathogens which provides integrity of dental pulp (Jiang, Zhang, Ren, Zeng, & Ling, 2006). On the other hand, inflammatory response to infections could inhibit cell proliferation of human dental pulp which disrupts the dynamic equilibrium of dental pulp (de Barros Silva et al., 2019). Also, TLR‐induced inflammation is the most important barrier to dental pulp reconstruction and complete tooth destruction (Colombo, Moore, Hartgerink, & D'Souza, 2014).

Toll‐like receptors (TLRs) are key components of the pattern recognition receptors (PRRs) family including 10 different functional receptors (TLR1‐TLR10) which recognize different pathogen‐associated molecular patterns (Jafari et al., 2018; Rusanen et al., 2017). The bacterial peptidoglycans, lipoproteins, and lipoteichoic acids of gram‐positive bacteria are identified by TLR2. Moreover, TLR3 detects double‐stranded RNA derived from viruses whereas, the lipopolysaccharide (LPS) of gram‐negative bacteria is identified by TLR4. TLR1, 2, 4, 5, 6, and TLR10 are the cell surface receptors, while TLR3, 7, 8, and TLR9 are expressed on intracellular endosomes (Taghavi et al., 2014). Downstream TLRs signaling is mainly recruitments myeloid differentiation factor 88 (MYD88) and activates nuclear factor kappa B (NFĸB), which in turn induces production of proinflammatory cytokines and chemokines (Ding & Liu, 2019; Gholamnezhadjafari et al., 2017).

The expression of *TLR4* on normal pulp tissues, odontoblast layer, and some pulpal vascular endothelial cells has well established (Jiang et al., 2006). Moreover, RT‐PCR and flow cytometry have shown high expression of *NOD1* (nucleotide‐binding oligomerization domain), *Nod2*, and *TLR2*, but not *TLR4*, in human dental pulp fibroblasts (Hirao et al., 2009). TLR4 ligation by LPS induces inflammatory response via MyD88, NF‐kB, mitogen‐activated protein kinase (MAPK) pathways and interleukin‐8 (IL‐8) production in dental pulp stem cells (He et al., 2013). Also, Wenxi He et al have shown that TLR4 engagement increased the expression of decorin in odontoblast cells through activation of MYD88, NF‐kB, and MAPK signaling pathways (He et al., 2012). An experimental study has reported that the expression of *TLR2* was 30‐fold higher than the *TLR‐4* in murine dental pulp tissue (Mutoh, Tani‐Ishii, Tsukinoki, Chieda, & Watanabe, 2007). On the other hand, inflamed pulp tissues from mice with severe combined immunodeficiency (SCID) have shown a significantly increased expression of *TLR‐2* and *TLR‐4* (Mutoh, Watabe, Chieda, & Tani‐Ishii, 2009). Furthermore, the expression of vascular endothelial growth factor (*VEGF*) in odontoblast‐like cells is upregulated by bacterial endotoxins via TLR4 signaling pathways (Botero, Mantellini, Song, Hanks, & Nör, 2003; Telles, Hanks, Machado, & Nör, 2003). Interestingly, increased expression of TLR4 by zoledronic acid (ZA), could stimulate the expression of inflammatory genes and induces bone makers in the rat dental pulp (de Barros Silva et al., 2019).

Although many studies have established the expression and function of TLR molecules in the dental pulp tissue, the detailed molecular mechanisms of TLRs expression and regulation of immune responses by TLRs engagement in opened and closed apex teeth are poorly understood. Therefore, this human study aimed to identify the main cell surface (TLR2, TLR4) and intracellular (TLR3) toll‐like receptors in opened and closed apex teeth.

## 2 MATERIALS AND METHODS

This study was approved by the Local Ethics Committee at Royan Institute (Approved number: EC/90/1072), and written informed consent was obtained before the collection of teeth samples, according to the Royan Institute Declaration.

Human dental pulp tissues were derived from extracted healthy teeth obtained from 20 orthodontic patients (10 opened apex and 10 closed apex premolar tooth) aged 14–21 years. The opened apex teeth were immature with opened apex (>2.5 mm of apical radiographic diameter), and no systemic health problems. The tooth was immediately immersed in a physiological solution and transferred to the laboratory for a genomic approach to identify *TLR2* (OMIM accession number 603028, NCBI NM_001318787.2), *TLR3* (OMIM accession number 603029, NCBI NM_003265.3), and *TLR4* (OMIM accession number 603030, NCBI NM_138554.5).

### 2.1 Human pulp tissues collection

To reveal the pulp chamber, the tooth surfaces were cleaned and cut around the cementum‐enamel junction using sterilized dental fissure burs. Then, pulps were gently removed and quickly placed in RNAlater (Ambion) at −70°C until processed for RNA isolation.

### 2.2 RNA extraction, cDNA synthesis, and qPCR

The pulp tissues were removed from RNA later and dissolved in 1 ml TRIzol (Sigma) to extract RNA, according to the TRI reagent standard protocol supplied by the manufacturer. To remove genomic DNA contamination from the samples, total RNA was treated using DNase I (Revert Aid Minus Fermentase Kit, Germany) and the random primers were used for the first‐strand cDNA synthesis.

First‐strand cDNA was synthesized using the RNA extracted from the pulp tissues as a template, the reverse and forward primers for *TLR2–4* (Table 1), oligo(dT) and dNTP mix using a kit purchased from Invitrogen. Also, Β‐actin housekeeping gene was used as a reference gene to ensure the reliability of the process and transcription analysis. The amplification was performed for 40 cycles at 95°C for 30 s, 59°C–61°C for 30 s, and 72°C for 30 s (Mönkkönen et al., 2007).

**TABLE 1:** Sequence of primers

Gene	Forward primer (5′‐3′)	Reverse primer (5′‐3′)	Product size (bp)
*TLR2*	TCGGAGTTCTCCCAGTTCTCT	TCCAGTGCTTCAACCCACAA	175
*TLR3*	GTATTGCCTGGTTTGTTAATTGG	AAGAGTTCAAAGGGGGCACT	156
*TLR4*	CGTGGAGACTTGGCCCTAAA	TTCACACCTGGATAAATCCAC	301
*B‐actin*	CAAGATCATTGCTCCTCCTG	ATCCACATCTGCTGGAAGG	90

Quantitative PCR was performed using an ABI. Briefly, SYBR Green real‐time PCR master mix (each reaction contained dNTPs, Platinum Tag DNA polymerase, forward and reverse primers and water to a final volume of 25 μl) was added to the PCR plate. Each sample was run in triplicate. Then, the PCR process was performed in the following conditions: 50 cycles at 95°C (the 30 s), 59°C–61°C for 30 s (different TLRs with different temperatures), and 72°C for 30 s.

The results from quantitative PCR (Threshold cycle value) were normalized against human β‐actin. The fold change was calculated as FC = 2−ΔΔCT. The normalized expression values were analyzed using the ANOVA test and results were determined as mean ± *SEM*, a significance level was set at *p* < .05.

### 2.3 Western blot analysis

Protein expression of TLRs 2–4 of pulp tissue was analyzed by Western blot. Protein was extracted from all dental pulp tissues and its concentration was determined using spectrophotometry. Briefly, the samples were snap‐frozen by liquid nitrogen and stored at −80°C. Next, microtubes were inserted on ice cubes, then 200 µl lysis buffer was added using the Qproteome Mammalian protein prep kit (Qiagen). The microtubes were homogenized and left on ice for 1 hr. Then, they were centrifuged with 1200 rpm at 4°C for 20 min. The supernatant was transferred to 1cc microtube and the concentration of total protein was determined using the Bradford protocol. Briefly, a series of standard (0, 250, 500 µg/ml) was prepared. 100 µl of each sample and standards were added to 5 ml of Coomassie Blue. All tubes (20 samples) were incubated for 5 min. Protein concentration was measured with a spectrophotometer at 595 nm wavelength.

The samples were passed through gel electrophoresis. Concurrently, separating gel (6%) and stacking gel (10%) were prepared, and all samples were transferred to prepared wells on stacking gels. To decrease errors during transferring, the first well had been left empty and the second one was filled by 10 µl protein marker, and followed by samples (15 μl) into each well. The samples are then transferred to PVDF membranes using a transfer buffer for 90 min. The nitrocellulose membrane was taken out, and protein transferring was checked using Panswa S.PVDF. First, the paper was washed using distilled water until the bands appeared. Then, the membrane was blocked with 5% skim milk in TBST for 1 hr at room temperature. The primary Ab anti‐TLR2–4 (Invitrogen) in 5% bovine serum albumin was added and incubated overnight at 4°C on a shaker. The membrane was washed three times with TBST and secondary antibodies (Invitrogen) in 5% skim milk in TBST were added and incubated for 1 hr. Also, the membrane was rinsed three more times and the final picture was recorded in a dark room with electrochemiluminescence (ECL) and radiographic film. Then the results were analyzed with image processing software (Image J version 1.8.0‐112).

## 3 RESULTS

### 3.1 Quantitative PCR and RT‐PCR

**FIGURE 1:**
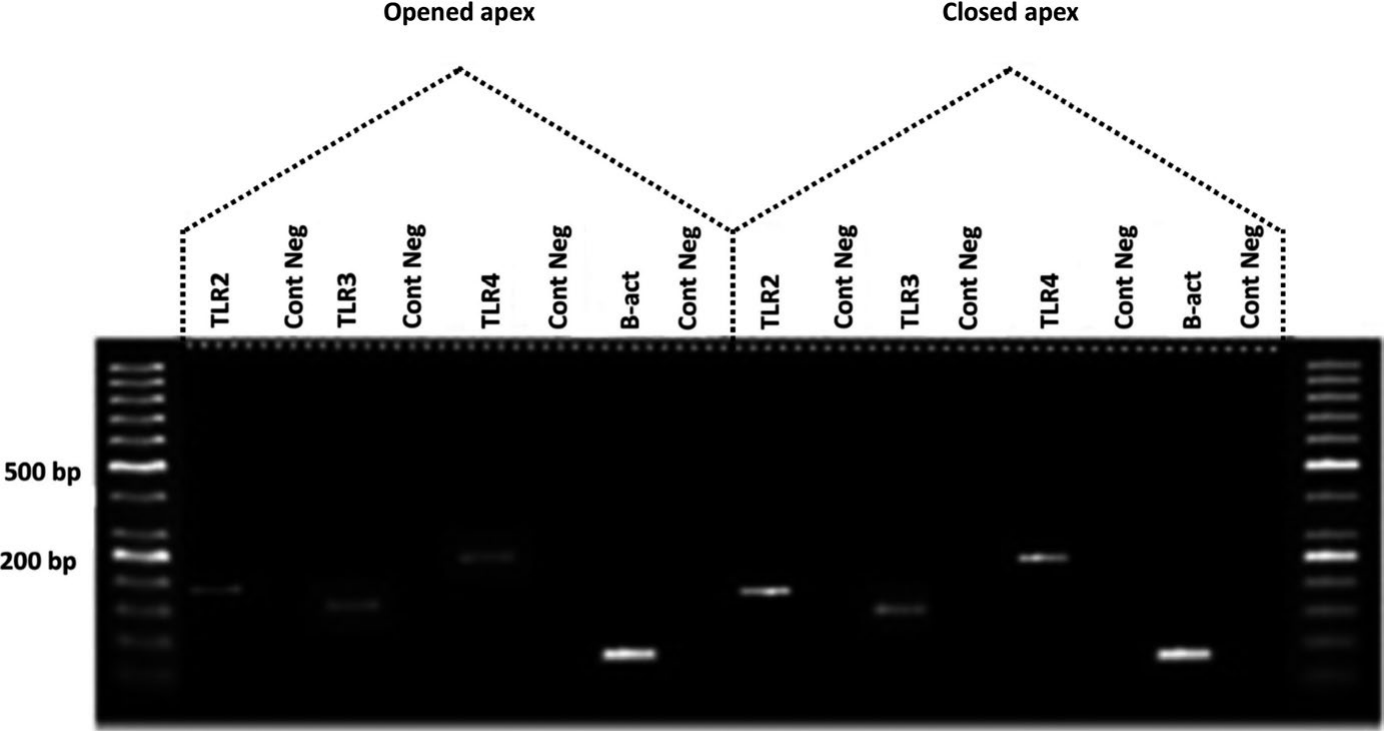
Agarose gel electrophoresis of the TLR2 *(OMIM accession number 603028, NCBI NM_001318787.2), TLR3 (OMIM accession number 603029, NCBI NM_003265.3) and TLR4 (OMIM accession number 603030, NCBI NM_138554.5)* genes in human Opened and closed apex dental pulp tissues of different teeth. Each pair of primers produced a specific product with the specific predicted size in each sample. There was no product amplified in control negative. *β-actin*=housekeeping gene

Gel electrophoresis was performed for mRNA expression of *TLR2–4* in opened and closed apex samples and results are presented in Figure 1. The product amplification was the predicted size for a particular gene. In the control negative, there was no product amplified, which indicated the absence of genomic DNA contamination.

**FIGURE 2:**
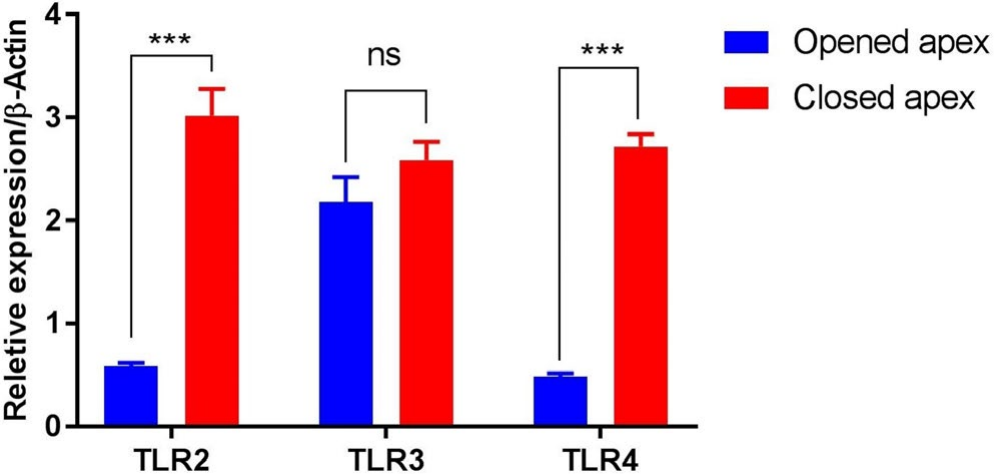
Mean ± *SEM* of normalized expression values for toll‐like receptor TLR2 (OMIM accession number 603028, NCBI NM_001318787.2), TLR3 (OMIM accession number 603029, NCBI NM_003265.3) and TLR4 (OMIM accession number 603030, NCBI NM_138554.5) genes in human dental pulp tissues of opened and closed apex teeth. The significance level was set at *p* < .05

The gene expression profiles of *TLR2* (OMIM accession number 603028, NCBI NM_001318787.2), *TLR3* (OMIM accession number 603029, NCBI NM_003265.3) and *TLR4* (OMIM accession number 603030, NCBI NM_138554.5) for tow studied groups are shown in Figure 2. *TLR2* and *TLR4* showed a significantly higher expression in closed apex premolar teeth compared to opened apex ones. On the other hand, no significant difference was detected in the relative expression of *the TLR3* between opened and closed apex premolars.

### 3.2 Western blotting

**FIGURE 3:**
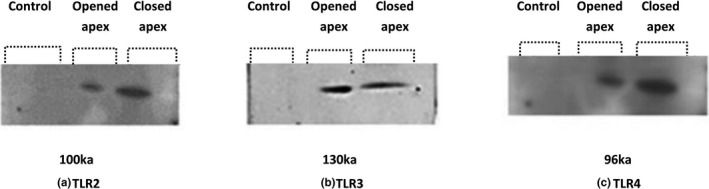
Protein analysis of TLR2–4 in the human dental pulp of open apex compared to closed apex in premolars using Western blot (a–c, respectively)

Western blotting results are demonstrated in Figure 3. There was a higher expression of proteins for TLR2 and TLR4 in closed apex premolars compared to the open apex. However, no significant difference was obtained between open and closed apex premolars for TLR3.

## 4 DISCUSSION

In this study, the effect of maturation of the apex of dental root on the expression of *TLR2–*4 genes and proteins was investigated. RT‐PCR and real‐time PCR were used as molecular methods to detect and quantify the gene expression of *TLR2–4*. Also, the Western blotting method was used to detect TLRs2–4 proteins.

Initial sensing of infection in the tooth is mediated by the PRRs of odontoblasts and leads to a significant increase in the level of inflammatory mediators cascade (Bianchi, 2007).

During tooth development, such tissues as bone and blood vessels undergo degradation, regeneration, and repair, which may lead to an increase in DAMPs (e.g., HMGB1) and more expression of TLRs molecules.

The results of the present study revealed that the expression of *TLR2–4* was mainly higher in mature teeth than those in the immature group. However, *TLR3* mainly remained intact compared to *TLR2* and *TLR4*. Growing evidence suggests that TLR3 is present both in immune and nonimmune cells and has a dual function in regulating the balance between inflammation and disease on the one hand and immune tolerance and inflammatory responses on the other (O'Neill, Golenbock, & Bowie, 2013). It is well established that TLR3 plays a positive role in the absence of infection (Heath & Carbone, 2013). Therefore, it can be concluded that its presence is almost independent of the quantitative amount of antigens. Moreover, the oral cavity is home to a variety of bacteria and viruses and these microorganisms are identified by PRRs, which leads to antibacterial and antiviral immune responses. Consistent with the results of the present study, a previous study reported the expression of *TLR3* on dental pulp fibroblasts that can induce TLR‐mediated inflammatory signals following ligation by specific agonists (Staquet et al., 2008).

The results of this study showed a significantly increased expression of *TLR2* and *TLR4* in closed apex compared to opened apex mature dental pulp. However, Hirao K et al have reported that *TLR4* is not expressed on the surface of dental pulp fibroblasts (Hirao et al., 2009).

Apical closure occurs 3 years after human tooth eruption; thus, if avulsion, intrusion, and extrusion occur during this time, it may result in cutting off the apical blood supply and rupture of nerve bundles and interruption of dentinogenesis (Torabinejad & Turman, 2011). The results of this study confirmed that the expression of *TLR2, TLR3*, and TLR4 is different in dental pulp with open and closed apex. Also, it was concluded that the expression of TLRs molecules in dental pulp tissue was associated with apex maturation of human teeth. The new results support the hypothesis that treatment of ligation of TLR4 on mice‐derived MSCs leads to the survival of MSCs and the release of vascular endothelial growth factor (Yao et al., 2009). Dental pulp tissue engineering is focused on the regulation of VEGF to increase MSCs survival via changes in TRLs' expression to improve the success of revascularization of the traumatized teeth (Gonçalves et al., 2007). Hence, the result from this study showed that gene expression of *TLRs* was heterogeneous in dental pulp with open and closed apex which leads to improving our knowledge of dental pulp tissue in the different teeth.

## CONFLICT OF INTEREST

There is no conflict of interest regarding the publication of this study.

## AUTHOR'S CONTRIBUTION

R.J and R.A conceived of the presented idea. F.P.H, F.S, and Z.C contributed to the design and implementation of the research. S.A, L.S, and K. N contributed to the analysis of the results. R.J and R. K wrote the manuscript with support from R. A.

## Data Availability

The datasets generated during this study are available on request from the corresponding author, Reza Aflatoonian.
